# In Vitro Antibacterial Effect of Probiotics Against Bacterial Isolates from Diabetic Foot Ulcers in Ain Shams University Hospitals

**DOI:** 10.1007/s12602-026-10981-4

**Published:** 2026-04-02

**Authors:** Mariam Tarek Eltoukhy, Basma Sherif, Atef Abdel Hameed Desouki, Samar Saad Rashad

**Affiliations:** 1 Clinical Pathology Department, Faculty of Medicine, Ain Shams University, Cairo, 11734 Egypt; 2 Vascular Surgery Department, Faculty of Medicine, Ain Shams University, Cairo, Egypt

**Keywords:** Antibacterial effect, Diabetic foot ulcers, Probiotics, *Lactobacillus*

## Abstract

Diabetic wound infections often develop as a result of multi-drug resistant (MDR) bacterial infections, creating a major therapeutic challenge. Probiotics are known to produce antimicrobial compounds with potential antibacterial effect. The aim of our study is to assess the antibacterial effect of different *Lactobacillus* strains against pathogens of diabetic foot ulcers (DFUs). 150 wound swabs were collected from inpatients and outpatients attending emergency room, Vascular Surgery and General Surgery Departments at Ain Shams University Hospitals who were suffering from DFUs. Bacterial isolates were identified by standard microbiological methods. The antibacterial activity of probiotics was assessed using well diffusion method. From the 150 wound swabs, 204 Gram-negative (GN) and Gram positive (GP) pathogens were isolated. Klebsiella species (spp.) were the most frequent bacteria (61; 29.9%) isolated from diabetic foot ulcers. Total carbapenem resistant (CR) isolates represented (64.9%). Lactobacillus plantarum exhibited notable antibacterial effects against both GN and GP isolates, the highest zone of inhibition (ZOIs) among GP was against staphylococcus aureus (17 mm), while, among GN was against Klebsiella spp. (9.23mm). Gram-positive bacteria were generally more susceptible than Gram-negative bacteria. Diabetic foot infections (DFIs) are commonly caused by GN-MDR pathogens, particularly Klebsiella spp., with a high prevalence of carbapenem resistance. Despite widespread antibiotic resistance, Lactobacillus cell free supernatant (CFS) exhibited inhibitory activity against both GP and GN isolates. These findings support the potential role of probiotics as an adjunct antimicrobial strategy, owing to its promising antibacterial effect, particularly against Gram-positive bacteria.

## Introduction

Diabetes mellitus (DM) is a major global health problem and ranks among the leading causes of morbidity and mortality worldwide. Its incidence and prevalence continue to increase, resulting in substantial health and economic burdens. DM is a chronic metabolic disorder characterized by impaired carbohydrate metabolism due to insufficient insulin secretion, impaired insulin action, or both, leading to persistent hyperglycemia. The disease is classified into three main types: type 1 diabetes mellitus (T1DM), type 2 diabetes mellitus (T2DM), which is the most prevalent form, and gestational diabetes mellitus. Prolonged poor glycemic control is associated with the development of microvascular and macrovascular complications, contributing significantly to increased morbidity and mortality among affected individuals [1].

Diabetic foot ulcer (DFU) is a serious chronic complication of diabetes mellitus, affecting approximately 15–25% of individuals with diabetes during their lifetime. According to estimates by the International Diabetes Federation (IDF), DFUs affected between 9.1 and 26.1 million people with diabetes worldwide in 2015. The global incidence of DFU continues to rise, largely due to increased life expectancy among diabetic patients and the growing worldwide prevalence of diabetes mellitus [2].

According to the International Diabetes Federation (IDF), Egypt ranks ninth globally in diabetes mellitus prevalence, with an estimated 8,850,400 adults affected in early 2020, corresponding to a prevalence rate of 15.2%. Egypt is part of the Middle East and North Africa (MENA) region, where the IDF projects a substantial increase in the burden of diabetes, with the number of affected individuals expected to reach approximately 108 million by 2045. A significant proportion of cases remain undiagnosed, as nearly 40–50% of individuals with diabetes or prediabetes are unaware of their condition. In Egypt, diabetes mellitus represents a leading cause of chronic kidney disease, visual impairment, lower-limb amputations, cerebrovascular accidents, and acute coronary syndromes [3].

Unfortunately, foot ulcers and amputations are very common in developing countries, infection of foot ulcers appears in more than half of all cases which requires hospitalization and 20% of lower extremity infections will lead to amputation [4].

Current guidelines for the management of severe diabetic foot infections (DFIs) recommend a comprehensive approach, including assessment of the need for surgical intervention, evaluation for the presence of peripheral arterial disease (PAD), and prompt management if PAD is detected. Empiric broad-spectrum antibiotics targeting both Gram-positive and Gram-negative bacteria, including anaerobes, should be initiated early. Subsequent antibiotic therapy must then be tailored according to clinical response and culture results [5].

Diabetic foot infections are classified as mild, moderate, or severe. Gram-positive bacteria, such as Staphylococcus aureus (S. aureus) and Beta-hemolytic Streptococci (βHS), are the most common pathogens in previously untreated mild and moderate infection. While severe, chronic, or previously treated infections are often polymicrobial, including gram-negative bacilli. Anaerobic pathogens are more commonly present in necrotic wounds and infections of the ischemic foot [6].

The emergence of antibiotic-resistant pathogens is occurring with increasing frequency, leading to a higher rate of treatment failure. Given the growing resistance to many commonly used antibiotics, there is an urgent need to develop alternative and safe therapeutic strategies to manage microbial infections. One promising approach involves utilizing probiotic bacteria derived from the human microbiome, which can inhibit the growth of pathogenic microorganisms and contribute to infection control [7].

Probiotics exert anti-inflammatory effects by scavenging reactive oxygen species, including superoxide and hydroxyl radicals, and by reducing the production of inflammatory cytokines, thereby potentially enhancing the wound healing process [8]. *Lactobacillus* species, a group of lactic acid–producing bacteria, play a natural role in controlling microbial populations in various environments. These lactic acid bacteria (LAB) exhibit antagonistic and inhibitory activities against pathogens, either by competing for nutrients or by producing bioactive antimicrobial compounds such as organic acids, hydrogen peroxide, acetylacetone, diacetyl, and bacteriocins [9].

Probiotics possibly exert a positive potential on the human body through these main mechanisms: competitive exclusion of pathogens, improvement in intestinal barrier functions, modulation of the immune system and production of neurotransmitters. Probiotics compete with pathogens for nutrients and receptor-binding sites, making their survival difficult. Probiotics modulates IgA and cytokine release, which limits growth of both Gram-positive and Gram-negative pathogenic bacteria. Nonetheless, these effects are strain-specific and dose-dependent [10].

## Materials and Methods

A total of 150 swab samples were aseptically collected on thioglycolate broth from inpatients and outpatients presenting with DFUs at the Emergency Room, Vascular Surgery, and General Surgery Departments of Ain Shams University Hospitals. The samples were obtained during the period from July 2024 to April 2025. Collected swabs were immediately transported to the Main Microbiology Laboratories at Ain Shams University Hospitals. Data collected from medical records were kept confidential, and the research had approval from the Ain Shams University Research Ethics Committee (Reg. No FMASU MS 404/2024, dated 11/6/2024).

### Type of Study

Cross sectional study.

## Inclusion Criteria

Diabetic patients more than 18 years old with infected foot ulcers.

### Exclusion Criteria

1. Duplicate samples from the same patient were excluded.
2. Diabetic patients less than 18 years old were excluded.

### Study Procedure

#### All Bacterial Isolates were Subjected to the Following

Identification by conventional techniques such as colonial morphology, gram stain characteristics and biochemical reactions [11].

Antimicrobial susceptibility testing was performed by disk diffusion method according to the Clinical and Laboratory Standards Institute guidelines (CLSI), 2024 [12].

Storage in Tryptone Soya Broth with 15% glycerol at -80 °C.

Subculture on blood agar plates.

Evaluation of the antibacterial effect of *Lactobacillus* CFS was carried out using the agar well diffusion method.

### Bacterial Identification and Characterization

All bacterial isolates were identified by conventional microbiological methods; colonial morphology, Gram staining, and biochemical tests; as for gram positive, catalase, coagulase and DNase, bile esculin tests were performed. As for gram negative; triple sugar iron, urease, citrate, lysine iron agar, motility indole ornithine tests were performed. Following identification, isolates were subjected to antimicrobial susceptibility testing (AST) according to CLSI (2024) using the disk diffusion method. Identified strains were stored in Tryptone Soya Broth (Oxoid, UK) at − 80 °C until further analysis. For subsequent testing, isolates were subcultured on blood agar plates and incubated at 37 °C for 24 h. Quality control was ensured using reference strains: Staphylococcus *aureus* (*S. aureus)* ATCC 25923, Methicillin-resistant *Staphylococcus aureus* (MRSA) ATCC BAA-1062, *Escherichia coli (E. coli)* ATCC 25922, *Pseudomonas aeruginosa* ATCC 27853, and *Klebsiella* ATCC 700603.

### Preparation of *Lactobacillus* Cell-Free Supernatant (CFS)

The *Lactobacillus* strains used were provided by the National Research Center: *Lactobacillus rhamnosus* (*L. rhamnosus*) B-445 and *Lactobacillus helveticus* (*L. helveticus*) CNRZ-32 (NRRL, Illinois, USA), *Lactobacillus acidophilus* (*L. acidophilus*) (Chr. Hansen’s Lab., Denmark), and *Lactobacillus plantarum (L. plantarum)* (Faculty of Agriculture, Ain Shams University). Each *Lactobacillus* strain was cultured in De Man, Rogosa and Sharpe (MRS) broth (1000 mL) containing 10% inoculum (10⁸ CFU/mL) and incubated anaerobically at 37 °C for 48 h. The cultures were centrifuged at 6000 rpm for 15 min to remove cells, and the resulting supernatants were filtered through 0.22 μm syringe filters to obtain sterile CFS, which was stored in 15 mL sterile conical tubes until testing.

Evaluation of the Antibacterial Effect of *Lactobacillus* CFS was determined using the agar well diffusion method. All Bacterial isolates were standardized to a 0.5 McFarland suspension and inoculated uniformly onto Mueller–Hinton agar (MHA) plates. Wells (7 mm diameter) were aseptically created using a sterile cork borer. Four wells were filled with 100 µL of CFS from each *Lactobacillus* strain, and one well with sterile distilled water served as a negative control. Plates were left at room temperature to allow diffusion, followed by incubation at 37 °C for 24 h.

### Interpretation

**Fig. 1:**
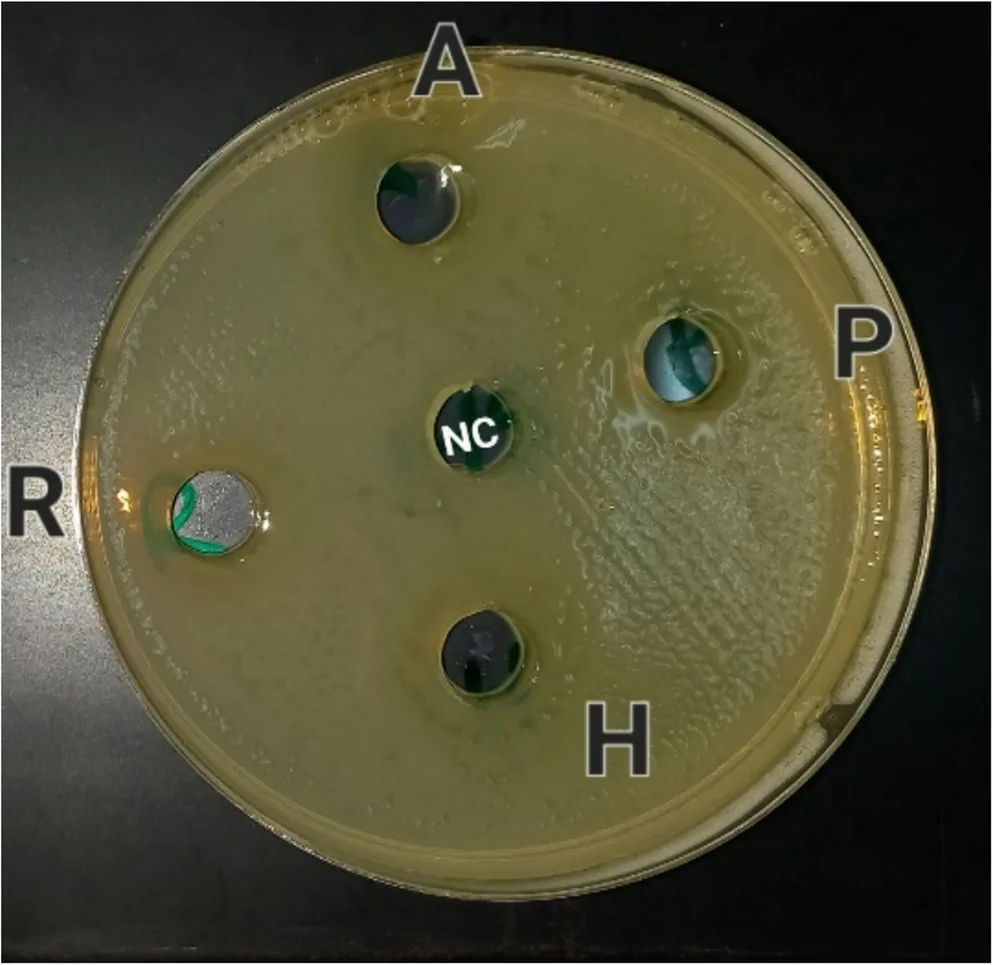
Negative antibacterial effect of *Lactobacillus* strains: MHA plate inoculated with *Klebsiella* spp. tested for susceptibility to CFS of *L. acidophilus* (A), *L. plantarum* (P), *L. helveticus* (H) and *L.rhamnosus* (R) The central well labeled “NC” represents the negative control. All wells showed 0 mm diameter for all *Lactobacillus* strains indicating no antibacterial activity against *Klebsiella*

**Fig. 2:**
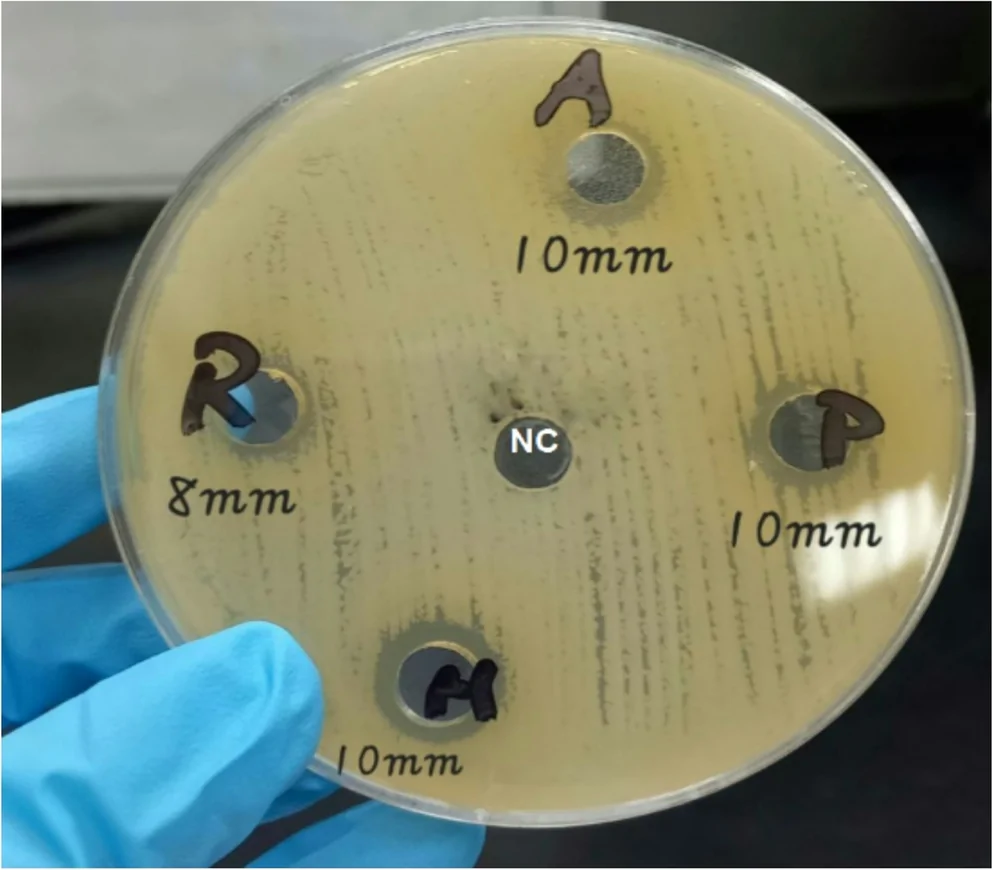
Weak antibacterial effect of *Lactobacillus* strains: MHA plate inoculated with *Klebsiella* spp. was tested for susceptibility to CFS of *L. acidophilus* (A), *L. plantarum* (P), *L. helveticus* (H), and *L.rhamnosus* (R). The ZOI measured 10 mm (+) for *L. acidophilus*, *L. plantarum*, *L. helveticus* and 8 mm (+) for *L.rhamnosus*

**Fig. 3:**
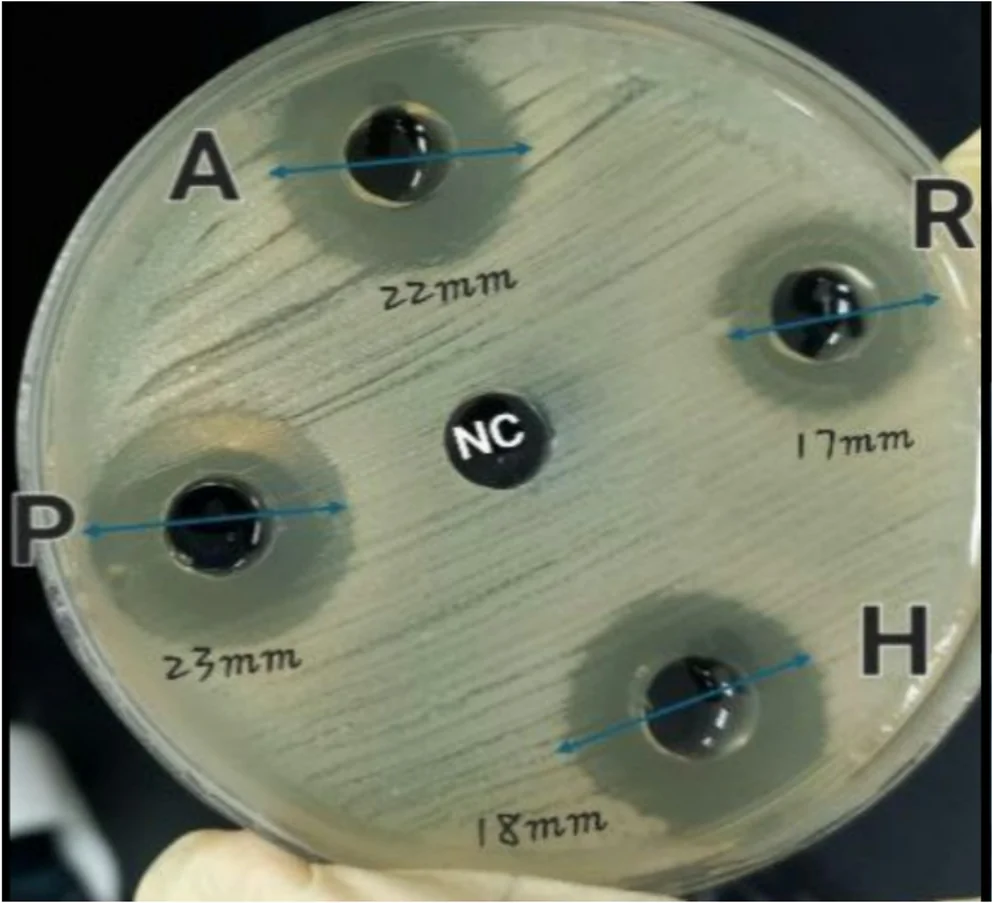
Intermediate & strong antibacterial effect of *Lactobacillus* strains: MHA plate inoculated with MRSA was tested for susceptibility to CFS of *L. acidophilus* (A), *L. plantarum* (P), *L. helveticus* (H), and *L.rhamnosus* (R). The ZOI measured 22 mm (+++), 23 mm (+++), 18 mm (++) and 17 mm (++) respectively

The zones of inhibition (ZOI) were measured in millimeters as demonstrated in Figs. 1, 2, and 3 and interpreted according to [13] as follows:

- 0 mm: no inhibition (−).
- 1–10 mm: low inhibition (+).
- 11–19 mm: intermediate inhibition (++).
- ≥ 20 mm: strong inhibition (+++).

### Statistical Methods

Data were analyzed using IBM SPSS Statistics for Windows, version 25.0 (IBM Corp., Armonk, NY, USA). Continuous variables were tested for normality using the Shapiro–Wilk test. Parametric data were expressed as mean ± standard deviation (SD), while non-parametric data were presented as median and interquartile range (IQR). Categorical variables were expressed as frequencies and percentages. The Kruskal–Wallis test was used to compare non-parametric variables among more than two groups, followed by Dunn–Bonferroni post hoc test for pairwise comparisons. Fisher’s exact test was applied to assess associations between qualitative variables when appropriate. A p-value < 0.05 was considered statistically significant.

## Results

### Demographic Data

The mean age of study cases was 55.6 ± 11.3 years old with males representing 58.7% of cases. Among the 150 patient samples, mixed bacterial growth was detected in 92/150 cases (61.3%). A total of 204 organisms were recovered, of which Gram-negative bacteria (GNB) predominated, accounting for 180 isolates (88.24%) followed by Gram-positive bacteria (GPB) with 18 isolates (8.82%) and Candida spp. with 6 isolates (2.94%). Table 1 summarizes culture results among the studied specimens and the most common antibiotic resistance patterns.

**Table 1:** Patient data and culture results among studied cases

				n.	%
**Type of Organism**	*Klebsiella* spp.			61	29.9%
	*Pseudomonas* spp.			40	19.6%
	*Proteus* spp.			28	13.7%
	*E. Coli*			28	13.7%
	*Acinetobacter* spp.			23	11.3%
	MRSA			14	6.9%
	Beta Hemolytic *Streptococcus*			2	1.0%
	*Enterococcus* spp.			2	1.0%
	*Candida* spp.			6	2.9%
**Infection type**	Mixed growth			92	61.3%
	Pure growth			58	38.7%
**Antibiotic susceptibility pattern**	MDR	CR-E		96	53.3%
		CR-*Ps*		21	11.7%
		CS-MDR		35	19.4%
		ESBLs		25	13.9%
	Non-MDR			3	1.7%

MRSA: Methicillin-resistant *Staphylococcus aureus*; CR-E: Carbapenem-resistant Enterobacterales; CR-*Ps*: Carbapenem-resistant *Pseudomonas*; ESBLs: Extended-spectrum Beta-lactamases; CS-MDR: Carbapenem-sensitive multidrug-resistant.

* CS-MDR: multidrug resistant GNB which tested sensitive to a carbapenem.

### Description of Antibiotic Susceptibility Pattern

Gram negative bacteria exhibit a high percentage of resistance to the majority of the tested antibiotics. Where *Klebsiella* spp., *Pseudomonas* spp., *E. coli*, *Proteus* spp. and *Acinetobacter* spp. were mostly resistant to AMC (100%), CIP (92.5%), DO & CTX (Both 100%), DO (92.9%), CIP (95.7%) respectively as shown in Table 2. As regard Gram positive bacteria; MRSA showed the highest sensitivity to VA and LZD being sensitive in all 14 (100%) tested isolates, while showed sensitivity in 21.4%, for CN and 57.1% for each of SXT, DA and E and intermediated sensitivity was noticed to 14.3% for LEV and DO. All tested βHS (two isolates) demonstrated complete sensitivity to AMP, LEV, E, DA, and LZD. Meanwhile, both *Enterococcus* isolates were sensitive to VA and LZD but resistant to all other antibiotics tested.

**Table 2:** Description of antibiotic susceptibility among sampled Gram-negative bacteria

		Organism									
		*Klebsiella* *N* = 61		*Pseudomonas* *N* = 40		*Proteus* *N* = 28		*E. Coli* *N* = 28		*Acinetobacter* *N* = 23	
		*N*	%	*N*	%	*N*	%	*N*	%	*N*	%
**AMC**	R	61	100%	*		20	71.4%	25	89.3%		
	I	0	0.0%			3	10.7%	2	7.1%		
	S	0	0.0%			5	17.9%	1	3.6%		
**SAM**	R	60	98.3%					26	92.9%	21	91.3%
	I	1	1.7%					2	7.1%	1	4.35%
	S	0	0.0%					0	0.0%	1	4.35%
**TZP**	R	56	91.8%	19	47.5%	12	42.8%	25	89.3%	20	87.0%
	I	0	0.0%	2	5.0%	1	3.6%	2	7.1%	1	4.3%
	S	5	8.2%	19	47.5%	15	53.6%	1	3.6%	2	8.7%
**FEP**	R	56	91.8%	32	80%	19	67.9%			20	87.0%
	I	1	1.6%	2	5.0%	0	0.0%			0	0.0%
	S	4	6.6%	6	15.0%	9	32.1%			3	13.0%
**CAZ**	R	58	95.1%	25	62.5%	21	75.0%	25	89.3%	20	87.0%
	I	0	0.0%	3	7.5%	0	0.0%	1	3.6%	0	0.0%
	S	3	4.9%	12	30.0%	7	25.0%	2	7.1%	3	13.0%
**CTX**	R	59	96.7%			20	71.4%	28	100.0%	21	91.3%
	I	0	0.0%			2	7.1%	0	0.0%	0	0.0%
	S	2	3.3%			6	21.5%	0	0.0%	2	8.7%
**IPM**	R			24	60.0%						
	I			2	5.0%						
	S			14	35.0%						
**MEM**	R	50	82.0%	24	60.0%	11	39.2%	14	50.0%	19	82.6%
	I	0	0%	0	0.0%	1	3.6%	3	10.7%	0	0.0%
	S	11	18.0%	16	40.0%	16	57.1%	11	39.2%	4	17.4%
**CN**	R	58	95.1%	38	95%						
	I	2	3.3%	0	0.0%						
	S	1	1.6%	2	5.0%						
**TOB**	R	54	88.5%	36	90.0%			25	85.7%		
	I	1	1.7%	3	7.5%			2	7.1%		
	S	6	9.8%	1	2.5%			1	4%		
**AK**	R	51	83.6%	32	80%	15	53.6%	25	89.3%	21	91.3%
	I	0	0%	5	12.5%	5	17.9%	0	0.0%	0	0.0%
	S	10	16.4%	3	7.5%	8	28.6%	3	10.7%	2	8.7%
**CIP**	R			37	92.5%	22	78.6%			22	95.7%
	I			0	0.0%	0	0.0%			0	0.0%
	S			3	7.5%	6	21.4%			1	4.3%
**LEV**	R	58	95.1%	36	90%	23	82.1%	27	96.4%	21	91.3%
	I	1	1.6%	1	2.5%	0	0.0%	0	0.0%	0	0.0%
	S	2	3.3%	3	7.5%	5	17.9%	1	3.6%	2	8.7%
**DO**	R	51	83.6%			26	92.9%	28	100.0%	19	82.61%
	I	1	1.6%			0	0.0%	0	0.0%	1	4.35%
	S	9	14.8%			2	7.1%	0	0.0%	3	13.04%
**SXT**	R	53	86.9%								
	I	0	0.0%								
	S	8	13.1%								

*S: sensitive; R: resistant; I: intermediate.

*Blank cells mean untested antibiotics either due to bacterial intrinsic resistance or due to lack of availability of antibiotic discs at time of antibiotic susceptibility testing of the strain.

### Description of *Lactobacillus*strains’ ZOI Among Each Sampled Bacterium

Table 3 summarizes the mean and median zone of inhibition (ZOI) diameters produced by each *Lactobacillus* strain against the tested bacterial isolates, the largest ZOI was observed in *Lactobacillus plantarum.*

**Table 3:** Description of *Lactobacillus* strains’ ZOIs among each sampled bacterium

	L. planatrum			L. acidophilus			L. helveticus			L. rhamnosus		
	Mean	±SD	Median	Mean	±SD	Median	Mean	±SD	Median	Mean	±SD	Median
***Klebsiella***	9.23 ^(+)^	6.34	11.0	9.02^(+)^	6.34	10.0	6.87^(+)^	6.57	8	5.77^(+)^	6.36	0
***Pseudomonas***	18.18^(++)^	4.49	18.0	16.70^(++)^	5.29	17.0	15.40^(++)^	6.21	16	15.28^(++)^	6.16	16
***Proteus***	16.54^(++)^	4.33	17.0	16.43^(++)^	5.70	18.0	14.79^(++)^	6.42	16	11.32^(++)^	8.03	15
***E. Coli***	10.57^(+)^	5.66	12.0	9.71^(+)^	5.79	11.5	8.46^(+)^	6.03	10	5.82^(+)^	6.23	4
***Acinetobacter***	12.00^(++)^	5.12	13.0	10.70^(+)^	4.98	12.0	8.13^(+)^	7.15	9	8.13^(+)^	6.31	9
**MRSA**	17.00^(++)^	2.96	0.17.0	15.00^(++)^	4.04	13.5	15.43^(++)^	4.24	15	14.00^(++)^	2.96	13
**βhs**	23.00^(+++)^	0.00	23.0	25.00^(+++)^	0.00	25.0	25.00^(+++)^	0.00	25	25.00^(+++)^	0.00	25
***Enterococcus***	18.00^(++)^	2	18.00	18.5^(++)^	3.5	18.5	15.5^(++)^	0.5	15.5	18.00^(++)^	3.16	18.00

MRSA: Methicillin-resistant *Staphylococcus aureus*; βhs: Beta Hemolytic *Streptococcus*; SD: standard deviation; *L. plantarum: Lactobacillus plantarum; L. acidophilus: Lactobacilus acidophilus; L. helveticus: Lactobacillus helveticus; L. rhamnosus: Lactobacillus rhamnous*

*(+) 1–10 mm: low inhibition, (++) 11–19 mm: intermediate inhibition, (+++) ≥ 20 mm: strong inhibition

### Comparison of *Lactobacillus* Effect Among MDR Groups

A highly significant difference in the size of ZOI was observed among Extended-spectrum Beta-lactamase (ESBLs), Carbapenem resistant Enterobacterales (CR-E), Carbapenem resistant Pseudomonas (CR-Ps) and carbapenem-sensitive MDR (CS-MDR) groups, with the highest ZOI observed in the CS-MDR group as shown in Table 4.

**Table 4:** Comparison of *Lactobacillus* antibacterial effect among MDR group

	*L*. acidophilus	*L*. planatrum					*L*. helveticus			*L*. rhamnosus		
	Mean	±SD	Median	Mean	±SD	Median	Mean	±SD	Median	Mean	±SD	Median
**ESBLs**	11.16	5.57	13	11.44	4.83	13	7.64	6.92	8	5.24	7.07	0
**CR-E/CR-Ps**	11.00	6.67	12	11.68	6.57	13	9.52	7.42	11	8.68	7.21	9
**CS-MDR**	16.40	5.80	17	17.57	5.25	18	15.17	5.37	16	13.23	7.13	15
**P**	0.001	0.001					0.001			0.001		

SD: standard deviation; *L. plantarum: Lactobacillus plantarum; L. acidophilus: Lactobacilus acidophilus; L. helveticus: Lactobacillus helveticus; L. rhamnosus: Lactobacillus rhamnous*; ESBLs: Extended-spectrum Beta-lactamases; CR-E: Carbapenem-resistant Enterobacterales; CR-Ps: Carbapenem-resistant *Pseudomonas*; CS-MDR: Carbapenem-sensitive MDR.

* CS-MDR: multidrug resistant GNB which tested sensitive to a carbapenem.

## Discussion

In the present study, wound swabs were obtained from 150 diabetic patients. Mixed microbial growth was detected in 92/150 (61.3%), pure growth was detected in 58/150 (38.7%). Overall, 204 microbial isolates were identified from the 150 specimens, with GNB being predominant, accounting for 180/204 isolates (88.24%). Gram-positive bacteria (GPB) constituted 18/204 isolates (8.82%), while Candida spp. represented 6/204 isolates (2.94%). Among the Gram-negative isolates, *Klebsiella spp.* were the most prevalent (29.9%), followed by *Pseudomonas spp.* (19.6%), *Proteus spp.* (13.7%), *Escherichia coli* (13.7%), and *Acinetobacter spp.* (11.3%).

Comparable findings were reported by Baral et al. [14], who observed that Gram-negative organisms comprised 82.97% of 141 isolates, whereas GPB accounted for 17.02%. In their study, *Klebsiella* was the most frequently isolated pathogen, followed by *Proteus spp.*,* E. coli*,* Pseudomonas spp.*, and *Staphylococcus aureus*, with mixed infections detected in 61.33% of ulcer samples. Similarly, Abu-El-Azayem et al. [15] documented a higher proportion of GNB (75.4%, 89/118) compared with GPB (22%, 26/118). The most common isolates in their study were Klebsiella (24%), Proteus (17.8%), Pseudomonas (16%), and S. aureus (13.5%), while *Candida species* were recovered in 2.6% of cases.

In agreement with our results, Hefni et al. [16] reported a predominance of Gram-negative bacteria (67%) over Gram-positive organisms (30%). The leading Gram-negative pathogens were *Pseudomonas aeruginosa* (19.4%), *Klebsiella pneumoniae* (15.3%), and *Acinetobacter spp.* (10.2%), whereas *S. aureus* (10.2%), *Streptococcus pyogenes* (7.1%), and MRSA (7.1%) were the most frequently isolated Gram-positive bacteria.

In contrast, Macdonald et al. [17] reported differing results, where only 62% of 200 samples were culture-positive. Among these, monomicrobial infections were more common (62.1%) than polymicrobial ones (37.9%), and Gram-positive bacteria predominated (96.1%), with S. aureus being the most frequently isolated organism (84.4%). Likewise, Palomo et al. [18] found that Gram-positive bacteria constituted the majority of isolates (68.1%), whereas Gram-negative bacteria accounted for only 31.9%. Additionally, 13.7% of cultures in their study showed no microbial growth, a finding that differs from the results of the current study.

It has been reported that Gram-positive aerobic cocci are the predominant pathogens in Western countries, whereas Gram-negative bacteria are more commonly isolated in warmer regions, particularly in Asia and Africa [19]. These variations may be explained by differences in geographical location, patient comorbidities, and institutional infection control practices. Although our findings demonstrate a clear predominance of Gram-negative organisms, particularly *Klebsiella* and *Proteus* species, these discrepancies highlight the importance of regional epidemiological surveillance to inform appropriate empirical antimicrobial therapy [7].

In the present study, carbapenem-resistant Gram-negative bacteria (CR-GNB) were detected in 117/180 isolates (65%). Among these, CR-E accounted for 96/180 (53.3%), while CR-Ps constituted 21/180 (11.6%). These findings are in agreement with those reported by Saleem et al. [20], who demonstrated that more than half of the 200 clinical isolates examined were carbapenemase producers. The predominant organisms identified in their study included *Klebsiella spp.*, *Pseudomonas spp.*, *Acinetobacter baumannii*, and *Escherichia coli*.

Similarly, Kotb et al. [21] documented a substantial burden of CRE in Egypt, reporting that 54.1% of 1,598 Enterobacter isolates collected between 2011 and 2017 were carbapenem-resistant. The high prevalence of CRE observed in these studies reflects an alarming regional trend in Egypt and across the Middle East and North Africa (MENA), largely attributed to the inappropriate and excessive use of carbapenems. The increasing emergence of CRE poses a significant clinical challenge due to the limited availability of effective therapeutic options and its association with higher rates of morbidity and mortality [22].

Conversely, Abu-El-Azayem et al. [15] reported that extended-spectrum β-lactamase (ESBL) production was more frequent, detected in 45% of Gram-negative isolates (40/89), whereas CRE accounted for only 22.5% (20/89). Additionally, Orfali et al. [23] observed complete susceptibility of all tested microorganisms to meropenem. The higher rate of CRE identified in our study may be explained by differences in local antimicrobial prescribing patterns, particularly the widespread empirical use of carbapenems, which exerts selective pressure favoring the emergence and dissemination of carbapenem-resistant strains.

The present study demonstrated that GNB showed a low susceptibility rate to most of the tested antibiotics. Klebsiella spp. exhibited low susceptibility to nearly all antibiotics used, with susceptibility rates of 0%, 8.2%, 6.6%, 4.9%, 3.3%, 9.8%, 3.3%, and 13.1% for both AMC & SAM, TZP, FEP, CAZ, CTX, TOB, LEV, and SXT, respectively. The highest susceptibility was observed with MEM (18%), AK (16.4%), and DO (14.8%). These findings were partially consistent with Mashaly et al. [24]; however, their study reported that *Klebsiella* spp. showed 100% susceptibility to AK, followed by MEM (90.9%) and TZP (81.8%). Additionally, a study conducted at Kafr El Sheikh University Hospital by Mabrouk et al. [25] revealed that *Klebsiella* spp. showed the highest sensitivity to DO, which was consistent with our findings. In contrast, Karvande et al. [26] reported that 77% of *Klebsiella* isolates were sensitive to TZP. The high rate of antibiotic resistance observed in our study may be attributed to prolonged use of broad-spectrum antibiotics and previous hospitalization, which predispose patients to colonization and infection with antibiotic-resistant organisms [27]. Furthermore, increased antibiotic resistance may also result from the horizontal transfer of antibiotic-resistance genes [28].

In this study, *Pseudomonas spp.* showed highest susceptibility to TZP (47.5%), MEM (40%), and IPM (35%). These findings are consistent with Shah et al. [29], who reported sensitivity to MEM and TZP, and Farhan et al. [30], who identified IPM as the most effective antibiotic. Mohamed et al. [31] also found IPM (72%) and TZP (62%) to be most active against *Pseudomonas*, while Mabrouk et al. [25] reported MEM and TZP as the most effective agents. In contrast, Alhubail et al. [32] observed high susceptibility to non-carbapenem antibiotics, with rates of 87% to AK, 80% to CN, and 77% to CIP. These rates were substantially higher than those observed in the present study (7.7%, 5.1%, and 7.5%, respectively). Such discrepancies may result from differences in local prescribing practices, regional resistance trends, and selective pressure from frequent use of aminoglycosides and fluoroquinolones.

*Proteus spp.* demonstrated highest susceptibility to MEM (57.1%) and TZP (53.5%). These results are consistent with Mulla et al. [33], who reported similar sensitivity patterns, while Thabit et al. [34] identified MEM (54%) as the most effective antibiotic. Conversely, Liu et al. [35] observed that *Proteus* isolates were predominantly sensitive to AK (97.14%).

Susceptibility of *E. coli* to most tested antibiotics was generally low. The sensitivity rates observed were AMC (3.6%), SAM (0%), TZP (3.6%), CAZ (7.1%), CTX (0%), TOB (4%), LEV (3.6%), and DO (0%), with the highest sensitivity recorded for MEM (39.2%) and AK (10.7%). Similar patterns were reported by Mashaly et al. [24], who found low sensitivity of *E. coli* to multiple antibiotics but full susceptibility to MEM and AK. Mabrouk et al. [25] also noted that AK and carbapenems were the most effective agents against these isolates. In contrast, Li et al. [36] reported high sensitivity to nearly all antibiotics, including 100% susceptibility to TZP and TOB, which differs from the current observations. Abbasi et al. [37] similarly found that TZP showed the highest activity against E. coli (88.9%). Such variations in susceptibility patterns may arise from differences in local resistance profiles, antibiotic exposure history, and clinical environments.

*Acinetobacter spp.* demonstrated low susceptibility to nearly all tested antibiotics. Observed sensitivity rates were SAM (4.3%), TZP (8.7%), FEP (13%), CAZ (14.3%), CTX (8.7%), AK (8.7%), CIP (4.3%), LEV (8.7%), and DO (13%), with the highest sensitivity percentage recorded for MEM (17.4%). These findings are consistent with Khan et al. [38], who reported generally low susceptibility of *Acinetobacter* to major antibiotic classes. Similarly, another study by Khan et al. [39] found low sensitivity to all tested antibiotics, including carbapenems, with 11.6% of isolates sensitive to MEM. In contrast, a study from South China by Liu et al. [35] reported 100% susceptibility to AK; however, this unusually high rate may be explained by the small number of *Acinetobacter* isolates (*n* = 6) included in that study.

All MRSA and *Enterococcus* spp. isolates in our study were fully susceptible to VA and LZD which was consistent with previous studies [24, 40, 41]. Some studies, however, reported less uniform sensitivity. For instance, Liu et al. [33] observed a few MRSA strains resistant to VA among DFU samples analyzed using the VITEK-2 Compact system, although overall susceptibility remained high (98% for VA and 100% for LZD). Differences in sample size may explain this variation, as Liu et al. [35] included 137 *Staphylococcus aureus* isolates. Regarding *Enterococcus*, Liu et al. [33] analyzed 79 isolates and reported susceptibility rates of 98% to LZD and 95% to VA, which aligns with the current findings, though the present study shows slightly higher sensitivity, likely due to the small number of *Enterococcus* isolates tested (*n* = 2).

The small sample size of βHS isolates (*n* = 2) limited broad interpretation of the results. Both isolates, however, were fully susceptible to P, E, DA, VA, and LZD, suggesting preserved sensitivity to traditional agents commonly used for streptococcal infections. This susceptibility pattern aligns with global trends, as resistance to penicillin and glycopeptides has not been reported in previous studies [42]. In contrast, resistance to lincosamides, macrolides, and fluoroquinolones has been documented in other reports.

The increasing prevalence of antimicrobial resistance has prompted the exploration of probiotics as potential alternative therapeutic agents. This study assessed the antibacterial activity of four *Lactobacillus* strains—*L. acidophilus*,* L. plantarum*,* L. helveticus*, and *L. rhamnosus****—***against bacterial pathogens isolated from diabetic foot infections, using the agar well diffusion method.

All *Lactobacillus* strains showed relatively low antibacterial activity against *Klebsiella spp.*, with the highest zone of inhibition (ZOI) observed for *L. plantarum* (9.23 mm), followed by *L. acidophilus* (9.02 mm) and *L. helveticus* (6.87 mm). ***L***. *rhamnosus* exhibited the lowest activity, with a ZOI of 5.77 mm. Abdelhalim et al. [22] reported similar findings when testing Lactobacillus strains against 11 CRE *Klebsiella* isolates, with *L. plantarum* producing a ZOI of 9 mm for two isolates, *L. helveticus* showing moderate inhibition across all isolates (mean ZOI ~ 13 mm), and *L. rhamnosus* demonstrating inhibition with a mean ZOI of ~ 10 mm. In contrast, Sharma et al. [43] found that *K. pneumoniae* were resistant to the cell-free supernatant (CFS) of all tested Lactobacillus strains, with no detectable ZOI. Additionally, Abdelhalim et al. [22] observed no antibacterial effect of *L. acidophilus* against CRE *Klebsiella* isolates, which may be attributed to the small sample size.

Antibacterial activity of all *Lactobacillus* strains against *Pseudomonas spp.* was generally intermediate, with ZOI ranging from 11 to 19 mm. *L. plantarum* showed the greatest inhibition (18.18 mm), followed by *L. acidophilus* (16.7 mm) and *L. helveticus* (15.40 mm), while *L. rhamnosus* exhibited the lowest activity (15.28 mm). These findings are in line with Al-Malkey et al. [44], who reported ZOI of 32 mm and 25 mm for *L. rhamnosus* and *L. acidophilus*, respectively, against *Pseudomonas aeruginosa* from wound infections. Prabhurajeshwar and Chandrakanth [13] also noted an average ZOI of 17.75 mm for Lactobacillus strains, indicating intermediate antibacterial effects. Lower inhibition was observed in the study by Ibrahim et al. [45], with mean ZOI of 9 mm and 8.17 mm for **L.** *acidophilus* and *L. helveticus* using the disk diffusion method. Even smaller ZOI (~ 5 mm) were reported by Al-Gamal et al. [46] for *L. plantarum*,* L. helveticus*,* and L. rhamnosus*, likely due to differences in sample size, the source of *Pseudomonas* isolates (cheese samples), and methodology, as they tested Lactobacillus cells with the agar overlay method rather than CFS.

Antibacterial activity against *Proteus spp.* was generally moderate for all *Lactobacillus* strains, with ZOI ranging from 11 to 19 mm. *L. plantarum* produced the largest inhibition (16.54 mm), followed by *L. acidophilus* ***(***16.43 mm) and *L. helveticus* (14.79 mm), whereas *L. rhamnosus* exhibited the lowest activity (11.32 mm). Comparable findings were reported by Sharma et al. [43], who observed ZOI of 12–15 mm for *L. plantarum*. In a study by Hegazy et al. [47], CFS of *L. plantarum* and *L. rhamnosus* showed moderate antibacterial effects against *Proteus*, with ZOI of 16.3 ± 1.50 mm and 13 ± 1.41 mm, respectively. Dawoud and Salman [48] also noted substantial inhibition by *L. acidophilus*, with a mean ZOI of 18.65 ± 1.05 mm.

*E. coli* isolates exhibited generally weak susceptibility, with ZOI below 10 mm. The largest inhibition was observed for *L. plantarum* (10.57 mm), followed by *L. acidophilus* (9.71 mm) and *L. helveticus* (8.13 mm), while *L. rhamnosus* showed the lowest activity (5.82 mm). Ibrahim et al. [45] reported similar results, with mean ZOI of 7–7.67 mm across the strains. In contrast, Noori et al. [49] observed no inhibition by *L. plantarum* or *L. acidophilus*, and Sharma et al. [43] noted resistance of *E. coli* to CFS of *L. helveticus*,* L. plantarum*, and *L. rhamnosus*, possibly due to bacterial tolerance to low pH, weak acids, and hydrogen peroxide. Arrioja-Bretón et al. [50] reported intermediate antibacterial effects, with mean ZOI for *E. coli* ATCC 25922 of 14.06 ± 1.24 mm for *L. acidophilus*, 20.23 ± 2.03 mm for *L. plantarum*, and 17.15 ± 3.58 mm for *L. rhamnosus*.

All *Lactobacillus* strains showed moderate to weak antibacterial activity against *Acinetobacter spp.* isolates. The largest ZOI was observed for *L. plantarum* (12 mm), followed by L. acidophilus (10.7 mm) and both *L. helveticus* and *L. rhamnosus* (8.13 mm). Hegazy et al. [47] reported mean ZOI of 16 ± 1.15 mm for *L. rhamnosus* and 13.5 ± 0.58 mm for *L. plantarum* against *Acinetobacter*. Similarly, Shah et al. [51] observed inhibition zones of 13.0 ± 1.41 mm for L. acidophilus and 15.0 ± 1.41 mm for *L. plantarum* against *Acinetobacter baumannii*. In contrast, Halder et al. [52] reported strong inhibition of *Acinetobacter* by *L. rhamnosus* (20.33 ± 1.53 mm), while ZOI for L. acidophilus and *L. plantarum* were 13.67 ± 0.58 mm and 16 ± 2.16 mm, respectively, which aligns closely with the present findings.

Antibacterial activity against MRSA was intermediate for all *Lactobacillus* strains, with *L. plantarum* showing the highest ZOI (17 mm), followed by *L. helveticus* (15.43 mm), *L. acidophilus* (15 mm), and *L. rhamnosus* (14 mm). Testing for βHS and *Enterococcus* was limited due to small sample size. These results are consistent with Hegazy et al. [47], who reported ZOI of 15.3 ± 1.32 mm and 12.8 ± 1.20 mm for *L. plantarum* and *L. rhamnosus*, respectively, against *Staphylococcus aureus*. Arrioja-Bretón et al. [50] observed ZOI of 13.91 mm, 13.93 mm, and 18.87 mm for L. acidophilus, *L. plantarum*, and *L. rhamnosus*, respectively, while Hussein et al. [53] reported significant inhibition by *L. plantarum* CFS with ZOI of 20 ± 0.34 mm. Conversely, Noori et al. [49] found that *L. plantarum* did not inhibit any pathogenic *S. aureus*, and Ibrahim et al. [45] reported weak antibacterial activity, with ZOI of 7 mm (*L. acidophilus*), 8.5 mm (*L. helveticus*), 6.33 mm (*L. plantarum*), and 6.5 mm (*L. rhamnosus*).

## Conclusion

The most common pathogens in DFU infections were found to be multidrug-resistant Gram-negative bacteria, especially *Klebsiella* species with a high incidence of CRE. *Lactobacillus* strains, particularly *Lactobacillus plantarum*, showed significant antibacterial activity against both Gram-positive and Gram-negative isolates despite widespread antibiotic resistance. Measurable inhibitory effects were shown by the CFS of *Lactobacillus*, with varying effects on Gram-negative pathogens and the highest action against Gram-positive bacteria. While activity against *Acinetobacter*,* Klebsiella*, and *E. coli* was minimal, *L. plantarum* and *L. acidophilus* had the strongest suppression against *Pseudomonas* and *Proteus* isolates. These results imply that metabolites generated from *Lactobacillus* have encouraging antibacterial properties and could be used as supplemental treatment agents to treat resistant DFU infections.

## Data Availability

The authors declare that data supporting the findings of this study are available within the article and its additional files.
